# Efficient methods and readily customizable libraries for managing complexity of large networks

**DOI:** 10.1371/journal.pone.0197238

**Published:** 2018-05-29

**Authors:** Ugur Dogrusoz, Alper Karacelik, Ilkin Safarli, Hasan Balci, Leonard Dervishi, Metin Can Siper

**Affiliations:** 1 Computer Engineering Dept., Bilkent University, Ankara 06800, Turkey; 2 Computational Biology Program, OHSU, Portland, OR 97239, United States of America; Universite de Nantes, FRANCE

## Abstract

**Background:**

One common problem in visualizing real-life networks, including biological pathways, is the large size of these networks. Often times, users find themselves facing slow, non-scaling operations due to network size, if not a “hairball” network, hindering effective analysis. One extremely useful method for reducing complexity of large networks is the use of hierarchical clustering and nesting, and applying expand-collapse operations on demand during analysis. Another such method is hiding currently unnecessary details, to later gradually reveal on demand. Major challenges when applying complexity reduction operations on large networks include efficiency and maintaining the user’s mental map of the drawing.

**Results:**

We developed specialized incremental layout methods for preserving a user’s mental map while managing complexity of large networks through expand-collapse and hide-show operations. We also developed open-source JavaScript libraries as plug-ins to the web based graph visualization library named Cytsocape.js to implement these methods as complexity management operations. Through efficient specialized algorithms provided by these extensions, one can collapse or hide desired parts of a network, yielding potentially much smaller networks, making them more suitable for interactive visual analysis.

**Conclusion:**

This work fills an important gap by making efficient implementations of some already known complexity management techniques freely available to tool developers through a couple of open source, customizable software libraries, and by introducing some heuristics which can be applied upon such complexity management techniques to ensure preserving mental map of users.

## Introduction

Networks are widely used in modeling information. Especially with the recent rapid rise of the web and big data technologies, more and more software companies are making use of so-called semantic graphs to represent huge amounts of data and employing advanced data visualization techniques along with data mining and knowledge discovery methods [1].

**Fig 1:**
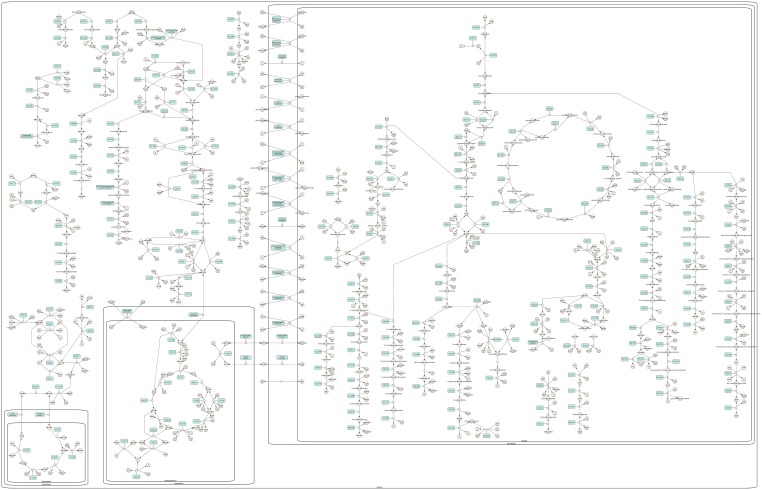
Large biological network example. Biological maps can be rather large as can be seen from this example map on plant central metabolism (http://sbgn.github.io/sbgn/examples).

**Fig 2:**
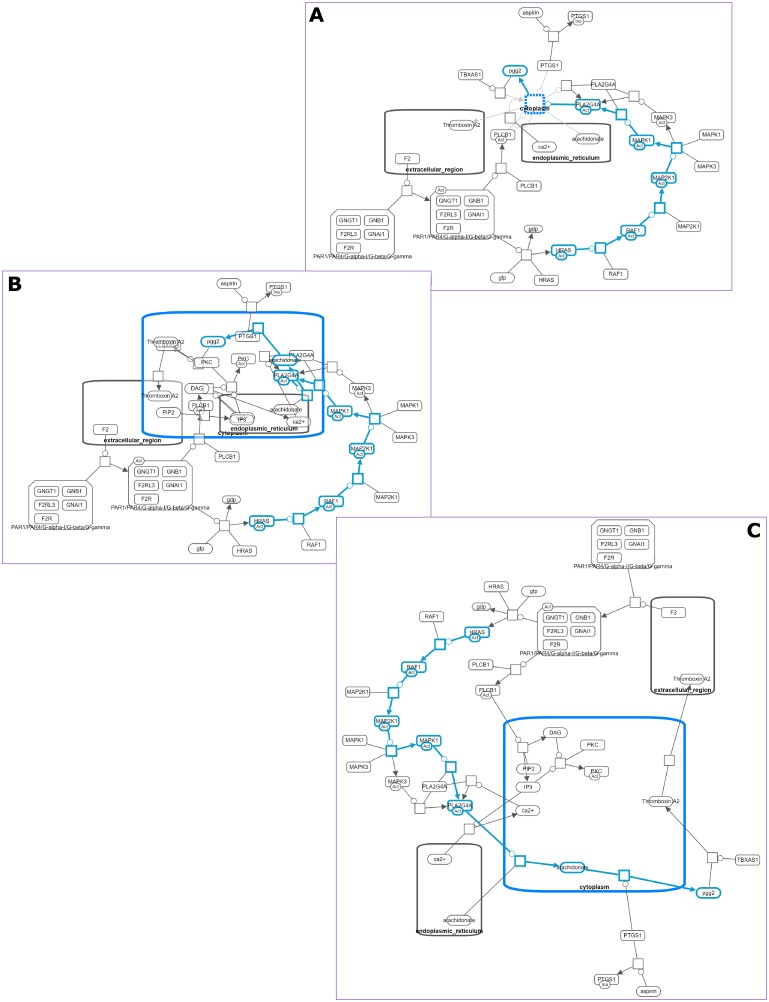
Losing mental map on expand operation. In a map with a certain path of interest highlighted for analysis, a compartment is collapsed to reduce complexity (A), the same map after the collapsed compartment is expanded but messed up layout is not adjusted (B), the same map after the collapsed compartment is expanded and layout is re-calculated from scratch; this moves the path of interest to a completely different part of the map, confusing the user (C).

**Fig 3:**
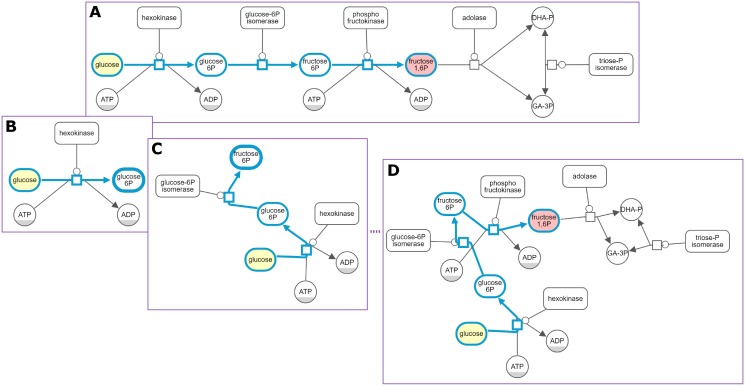
Losing mental map on unhide operations. In a map with a certain path of interest highlighted for analysis (A), processes currently out of focus are hidden to reduce complexity (B); subsequent unhide operations reveal hidden processes gradually, but since there is no specialized re-calculation of layout, parts of the map get tangled and the path of interest is oriented differently, ruining the user’s mental map (C,D).

Effective visual analysis of such large networks in numerous areas including systems biology (Fig 1) is only possible when interactive operations on these networks can be executed quickly. Unfortunately, most of these operations such as performing automatic layout or calculating a graph-theoretic property of a network do not scale well. Besides, even though it might be possible to render such large networks in its entirety on a computer display, resulting drawings are consistently overwhelming for the user, if not impossible to make any sense of. Hence, for navigating and visualizing such large networks, one needs to first reduce their complexity [2, 3]. Complexity management techniques range from simple rendering methods like panning, zooming and ghosting, to collapsing the compound nodes of a hierarchical network, to temporarily hiding unwanted parts of the topology. Even though existing complexity management methods are relatively straightforward to implement, their application frequently results in major changes in network topology, destroying existing layout of the network and user’s mental map of the underlying relational data (Figs 2 and 3).

Cytoscape.js [4] is an open source web based graph analysis and visualization library with a plug-in architecture (through so called *extensions*) for custom use.

Here, we present specialized methods to re-arrange networks upon complexity management operations, fixing the network layout and preserving the user’s mental map. In addition, we introduce two new libraries, which have quickly become two of the most popular Cytoscape.js extensions, especially in pathway visualization applications, to efficiently reduce the complexity of large networks via expand-collapse and hide-show operations.

### Background

A *graph* or a *network* is a representation of a set of objects, called *nodes*, where some pairs of objects are connected by links, called *edges*. An edge is said to be *incident upon* its source and target nodes; and source and target nodes are said to be *adjacent*. A *path* in a graph is a sequence of edges which connect a sequence of distinct nodes. A *tree* is a graph in which any two nodes are connected by exactly one path. A *rooted tree* is a tree with a countable number of nodes, in which a particular node is distinguished from the others and called the *root*.

**Fig 4:**
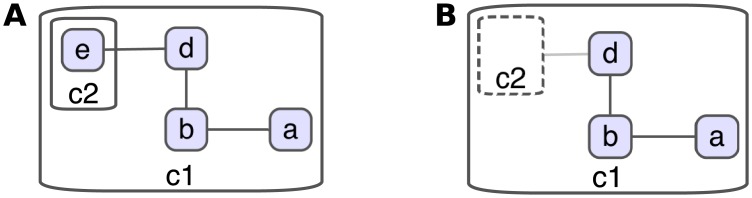
Compound graph example. A compound graph *C* = (*V*, *E*, *F*), where *V* = {*a*, *b*, *c*1, *c*2, *d*, *e*}, *E* = {{*a*, *b*}, {*b*, *d*}, {*d*, *e*}}, and *F* = {(*c*1, *a*), (*c*1, *b*), (*c*1, *c*2), (*c*1, *d*), (*c*2, *e*)}. The graph includes two compound nodes *c*1 and *c*2, and only one inter-graph edge {*d*, *e*}. *c*1 is the only node in root level (level 0), whereas nodes such as *c*2 and *a* are level 1 nodes (A). Same compound graph with *c*2 collapsed and original inter-graph edge {*d*, *e*} represented with a newly introduced meta edge {*d*, *c*2} (B).

A *compound* or *hierarchical* graph *C* = (*V*, *E*, *F*) consists of nodes *V*, adjacency edges *E*, and inclusion edges *F* [2]. It is required that the inclusion graph *T* = (*V*, *F*) is a rooted tree, and no adjacency edge connects a node to one of its descendants or ancestors. Compound nodes enable modeling complex structures through nesting when drawing graphs. An *inter-graph edge* is one with incident source and target nodes included in different compound nodes or owner graphs (Fig 4). For instance, recently developed Systems Biology Graphical Notation (SBGN) [5] makes exclusive use of compound structure to represent molecular complexes, sub-cellular locations, and sub-maps of a biological map.

A compound node is sometimes shown in a *collapsed* manner, particularly for complexity management purposes. In such cases, it’s customary to represent inter-graph edges with at least one end in the collapsed content with so called *meta edges* to keep relations intact (Fig 4).

Graphs are usually shown with a pictorial representation. A poor *layout* of a graph may confuse the user, whereas a well-organized one aesthetically pleases the users and improves their understanding of the underlying data. Criteria of a good layout is often subjective. However, generally accepted criteria include minimal number of edge–edge crossings, minimal drawing area, minimal total edge length and uniform edge length throughout the graph [6].

Force-directed layout algorithms (i.e., spring embedders) are the most popular and easiest to implement, where the basic idea is to simulate a physical system adhering to the basic laws of physics. Nodes are considered to be particles with the same electrical charge, repelling each other when they are too close, whereas the edges are represented with springs attracting their end nodes when they are too far [7]. The goal is to move particles iteratively to a stable state, where all forces cancel each other or the energy of the system is minimized. Most force-directed layout algorithms make use of a *temperature*, which is a special case of a more general technique called *simulated annealing* to force the algorithm to quickly reach a local minima of the energy function. CoSE is a force-directed layout algorithm that supports compound nodes [8].

**Fig 5:**
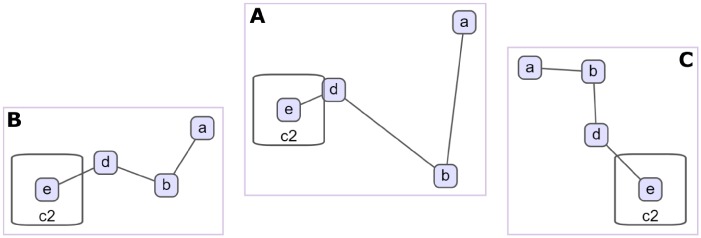
Incremental vs. static layout. A graph that needs a better layout due to changes in topology and/or geometry (A), the same graph after an *incremental* layout, where respective node positions are maintained (B), the same graph after a non-incremental (i.e., static) layout, during which previous node positions are ignored (C).

In interactive visualization applications, often times, the graph evolves over time, requiring a re-layout. Calculating a new layout, taking into account any available current positions, is said to be *incremental*, where the goal is “tidying up” the drawing without destroying the current respective positions of the nodes referred to as the user’s mental map (Fig 5).

The increase in the size of relational information to be visualized boosted the demand for more sophisticated complexity management techniques. A good deal of research has been conducted to solve the complexity management problem for large graphs [2, 9]. Some simply suggest *ghosting* or *hiding* unwanted details to later reveal on demand. Others make use of various types of lenses such as fisheye lenses [10] to focus on a user-chosen part of a large network, usually by making that part of the drawing larger compared to the rest. Some others describe how to create clusters based on a given data set with support for multiple views and nesting, and make use of expand-collapse operations to deal with complexity [11].

Abundant research on complexity management typically ignores implementation issues, not making any effort to preserve the user’s mental map as changes occur in both topology and geometry. This mental map can be preserved in different aspects: *orthogonal ordering*, *clusters* and *topology* [12, 13]. Orthogonal ordering is preserved if the horizontal and vertical orderings of nodes are maintained after an operation. Clusters are preserved by keeping nodes close in the updated layout if they were close in the original drawing. Finally, the topology is preserved if the layout of the graph after update is homeomorphism of the previous layout (i.e., preserves respective node positions). Other properties which are important to preserve for some applications include straightness of lines, orthogonality of lines parallel to the *x* and *y* axes, and relative sizes of nodes.

**Fig 6:**
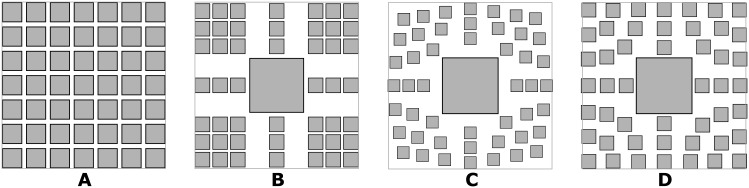
Scaling strategies to adjust layout. A grid of nodes (A), nodes scaled to preserve orthogonality when the node of interest (middle one) is enlarged (B), and nodes scaled to preserve proximity (clustering and topology, respectively) when the node of interest is enlarged (C and D, respectively) [13].

**Fig 7:**
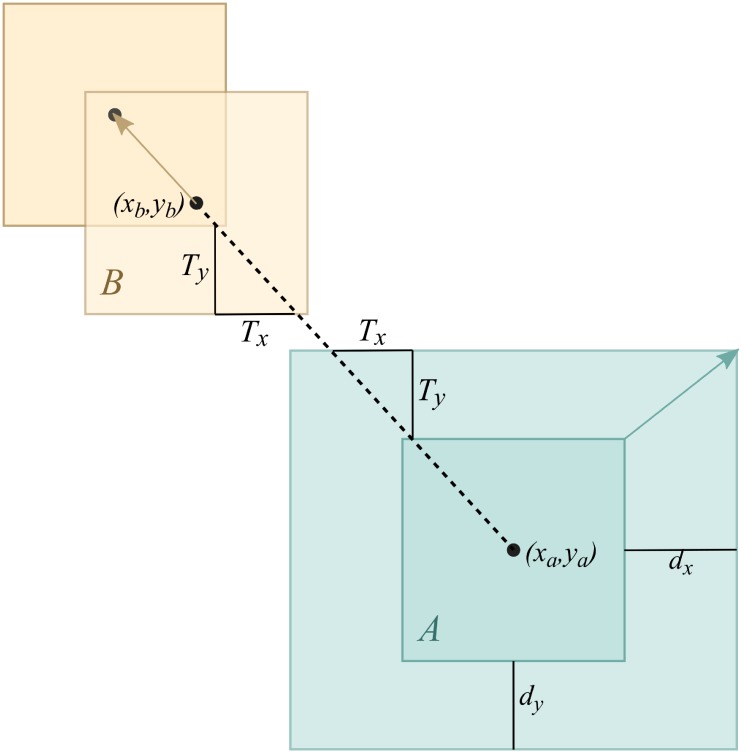
Calculations for preserving proximity. [13]. Node of interest *A* is expanded to be larger, keeping its center. This requires a neighboring node *B* to move in the direction from the center of *A* at (*x*_*a*_, *y*_*a*_) to center of B at (*x*_*b*_, *y*_*b*_) by as much as the change in the required space (*T*_*x*_, *T*_*y*_).

Storey et al. [13] discusses various layout adjustment strategies upon a node of interest changing dimensions (Fig 6). Among these strategies, use of proximity (aiming to preserve clustering and topology) seems to yield the best results in the context of expand-collapse operations. The idea here is to push out (pull in) surrounding nodes as much as the change in the required space. Hence, magnitude of the translation vector for each node is dependent on the relative location of that node and the scaled node (Fig 7).

Many tools have been developed in the past for visualization of all sorts of networks including biological ones [3, 14, 15]. Among these, Cytoscape.js [4] stands out as an open-source web based graph analysis and visualization library with a plug-in architecture through extensions for custom use, distributed under the MIT license. It was developed as a web based version of the popular application named Cytoscape [14], and quickly became a favorite choice of web based visual tool developers. It is currently in use by numerous applications from academia and industry. The library is compatible with modern technologies and libraries such as webpack, Node.js, and npm, and runs on all modern browsers.

Almost all network visualization tools support some form of automated layout. However, compound structures are occasionally supported by tools and almost never taken into account during automated layout [3, 16].

Tool support for complexity management operations on large networks is not very common. To the best of our knowledge (prior to this work) only one commercial visualization software by Tom Sawyer Software [17] embraces proper support for such operations. Other tools rarely support complexity management but without any layout adjustment. For instance, D3 [18], an open source visualization library, has some limited support for hiding a subtree of a tree structure and gradually revealing it. Furthermore, our libraries are the only free, open source complexity management libraries that can be customized for application-specific needs.

## Methods

### Adjusting layout upon an operation

As stated earlier, a major challenge upon application of complexity reduction operations on large networks is to keep the user’s mental map intact. This might require a customized layout algorithm for each complexity management operation. Towards this goal, we adapted and used methods described below.

#### Adjusting layout on collapse

**Fig 8:**
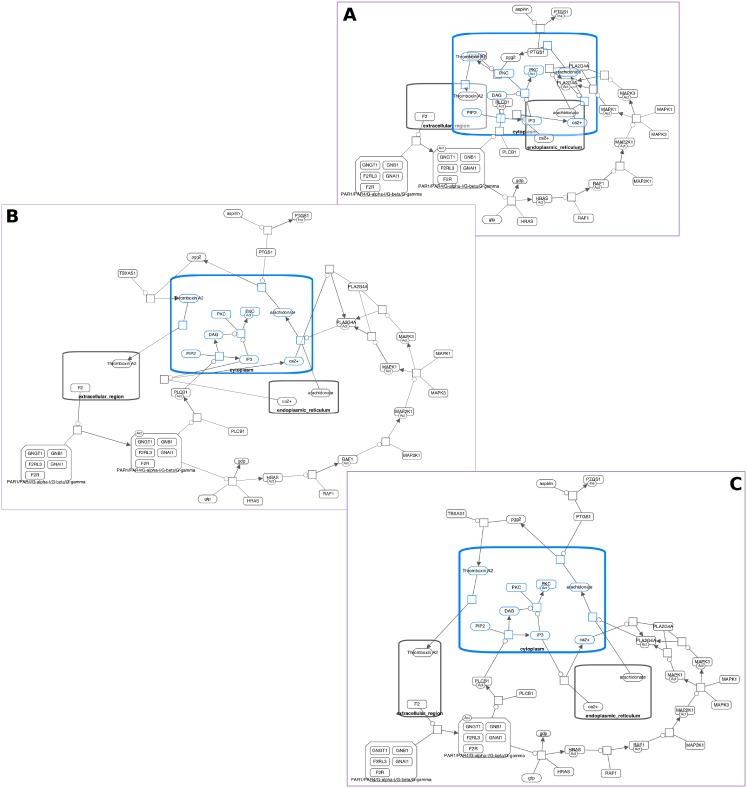
Adjust layout on collapse. An example of how applying incremental force-directed layout with a low initial temperature upon a collapse operation can predominantly keep respective positions of nodes while disposing of any newly introduced space around the collapsed node. A biological network (A) after a node of interest is collapsed (B), and additionally an incremental layout is applied as described (C).

A collapse operation always results in reduction in the dimensions of the node of interest, which, in turn, results in unnecessary space around this node (Fig 8). Hence, an incremental force-directed layout, where current node positions are used as initial positions should work just fine. One needs to make sure nodes are not allowed to move drastically however. Hence, starting an incremental layout with a *low* temperature should eliminate the unnecessary space around the node of interest introduced by the operation, while preserving the mental map.

#### Adjusting layout on expand

**Fig 9:**
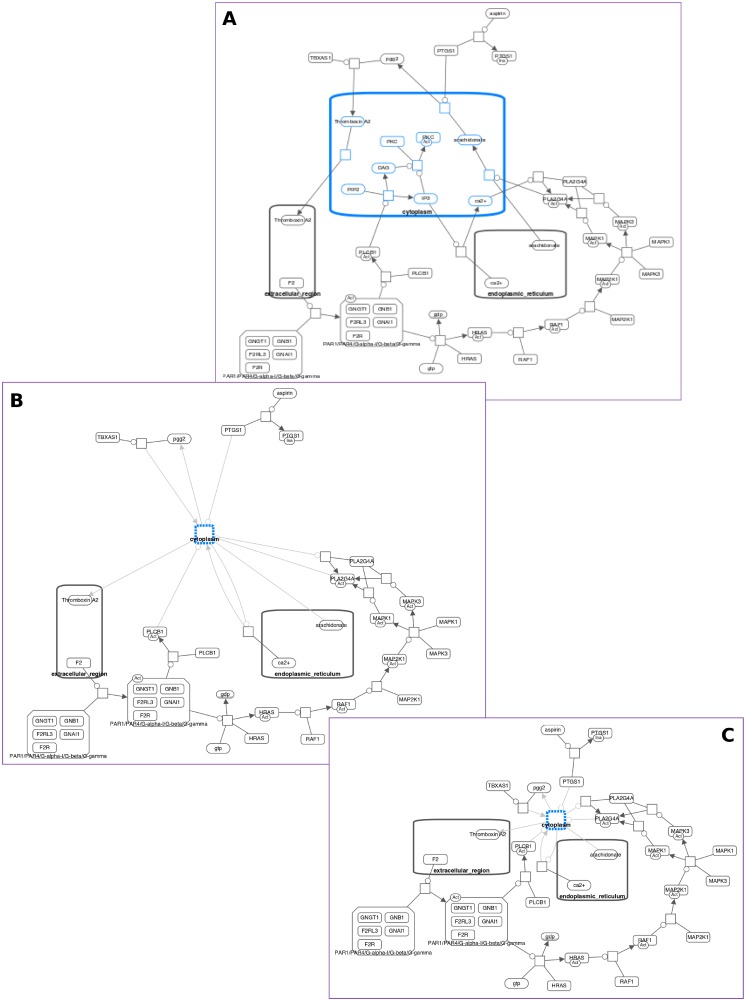
Adjust layout on expand. An example of how space proportional to the growth in expanded node needs to be opened up around the node before expand can be performed. For the graph in Fig 8.A, an expand is performed on the central node without any adjustment (A), when an expand is performed on the central node after an adjustment using a method by Storey et al. inspired by fisheye lens paradigm (B), followed by an incremental layout with low initial temperature to “polish” the layout (C).

Expand operation, on the other hand, almost always will result in an overlap of the node of interest with nearby nodes if enough space is not opened up prior to the operation (Fig 9). The method by Storey et al. [13] described earlier, which is inspired by the fisheye lens paradigm, will make enough room around the node to be expanded to accommodate the change in size. However, a final polishing step of an incremental layout with a low initial temperature can improve the drawing in terms of commonly accepted criteria of a good layout such as uniform edge length and minimal drawing area.

#### Adjusting layout on hide

Similar to the collapse operation, layout of a network with hidden parts can be adjusted by simply applying a specialized incremental layout. This should dispose of any unnecessary space introduced by the hide operation, while maintaining respective locations of nodes.

#### Adjusting layout on show

Adjusting layout upon a show (i.e., unhide) operation might not be straightforward however, especially when hidden content has neighbors in the network. This is due to the fact that after a certain part of a network is hidden, the layout of the remaining network might change drastically. In fact, even a simple translation of the entire drawing by the same amount will invalidate (i.e., destroy respective positioning) coordinates of the hidden content. Hence, unhidden neighbors of existing nodes need to be carefully placed back to the drawing without introducing any overlaps. For this, we devised a greedy algorithm to introduce hidden neighbors level by level *while* applying an incremental layout.

**Fig 10:**
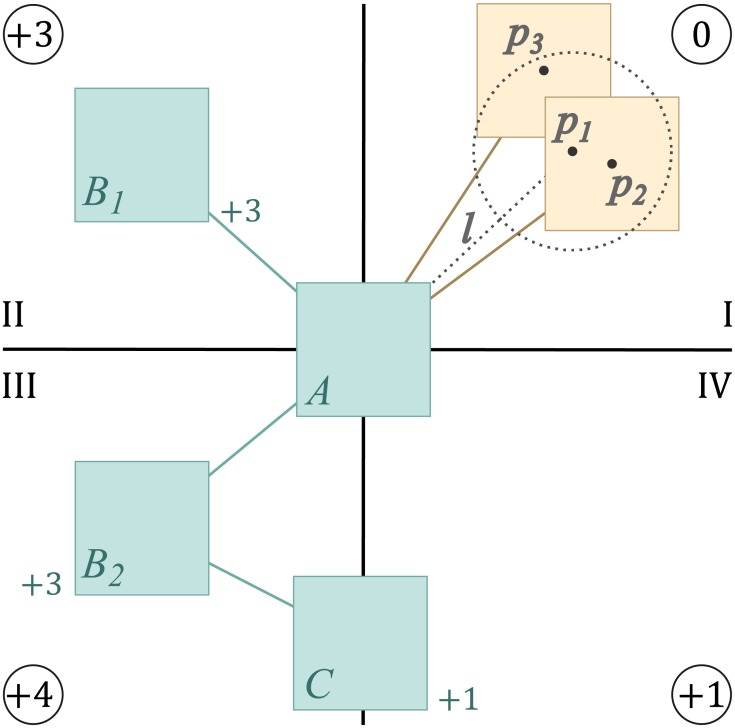
Heuristic for calculating location of unhidden neighbors. Each first (nodes *B*_1_ and *B*_2_) and second (node *C*) degree neighbor of node of interest (node *A*) contribute a score of +3 and +1, respectively, to the quadrant that they are in. Total score of each quadrant is circled. The quadrant with the least total score (quadrant I in this example) is chosen to place unhidden neighbors of the node of interest. The neighbors are placed to be “roughly” ideal edge length ($l=|\bar{A_{center}p_{1}}|$) away from the node of interest.

In order to determine the initial position of a newly unhidden node, we use a heuristic as sketched in Fig 10. Here, the idea is to split the neighboring space around the node with neighbors to be unhidden (i.e., the node of interest) into quadrants and scoring how crowded each quadrant is, using a simple metric. In order to be able to calculate this score quickly (in expected constant time), we only consider first and second degree (distance 1 and 2, respectively) neighbors of the node of interest, and assign a score of +3 and +1, respectively. We then determine the most available quadrant based on these scores and place unhidden neighbors *randomly* around the center of that quadrant with an approximate distance of *ideal edge length* from the node of interest. Once immediate neighbors of the node of interest are placed in this manner, several iterations of incremental layout are performed before a new level of neighbors of neighbors can be placed and integrated into the current drawing recursively. Unlike previous methods, where adjustment of layout is a post-processing step to the complexity management operation, with this method, layout adjustment *interleaves* with the complexity management operation.

### Handling meta edges

If not designed carefully, meta edge handling in expand-collapse operations can be rather costly in terms of both execution time and memory requirements. In order to achieve this, we came up with a robust and consistent mechanism, in which no meta edge is redundantly created (only one meta edge per original hidden edge) and resulting representation is independent of the order of expand-collapse operations. The only additional memory requirement besides the source and target of a meta edge is a pointer to the associated original edge.

As a result, expand-collapse operations can be performed as follows:

**algorithm** CollapseNode(CompoundNode c)

1 ig-edges ← {*e* = {*x*, *y*} | *x* is inside *c* ∧ *y* is outside *c*}

2 **for each** *e* = {*x*, *y*} ∈ ig-edges **do**

3  **if** *e* is an original edge **then**

4   create a meta edge *m* between *c* and *y*

5   *m*.*original* ← *e*

6  **else** //meta edge

7   *e*.reconnect(*c*,*y*) //*e*.*original* remains same

**algorithm** ExpandNode(CompoundNode c)

1 *G* ← *c*.*owner*

2 meta-edges ← {*m* = {*x*, *y*} | *m* is a meta edge ∧ (*x* = *c* ∨ *y* = *c*)}

3 **for each** *m* = {*x*, *y*}∈ meta-edges **do**

4  *e* ← *m*.*original*

5  *s* ← *e*.*source* //assume *c* to be source of *m*; other case is symmetric

6  **if** *s*.*owner* = *c* **then** //*s* is (immediately) in *c*

7   *t* ← *e*.*target*

8   **if** *t*.*owner* = *G* **then**

9    discard *m*

10   **else**

11    *m*.reconnect(*s*,*m*.*target*)

12  **else**

13   *c*_*s*_ ← compound node (immediately) in *c* ∧ (recursively) containing *s*

14   *m*.reconnect(*c*_*s*_, *m*.*target*)

The complexity of above algorithms is linear in the number of nodes collapsed (expanded) and the number of their associated edges. For the expand operation, however, there is an additional cost factor for the method that finds the highest level of a compound containing a particular node for each meta edge incident with the compound being expanded. This factor though, can be assumed to be a constant since the nesting depth is expected to be not very high and independent of network size in real-life networks.

## Libraries

We implemented aforementioned methods in JavaScript, making sure to meet the industry requirements for generality (works for undirected graphs from all domains), efficiency (performs well within an interactive tool), and extendibility (can be easily customized). These libraries, which were developed and packaged as Cytoscape.js extensions, are two of the most widely used ones among dozens of available extensions.

### Expand-collapse

**Fig 11:**
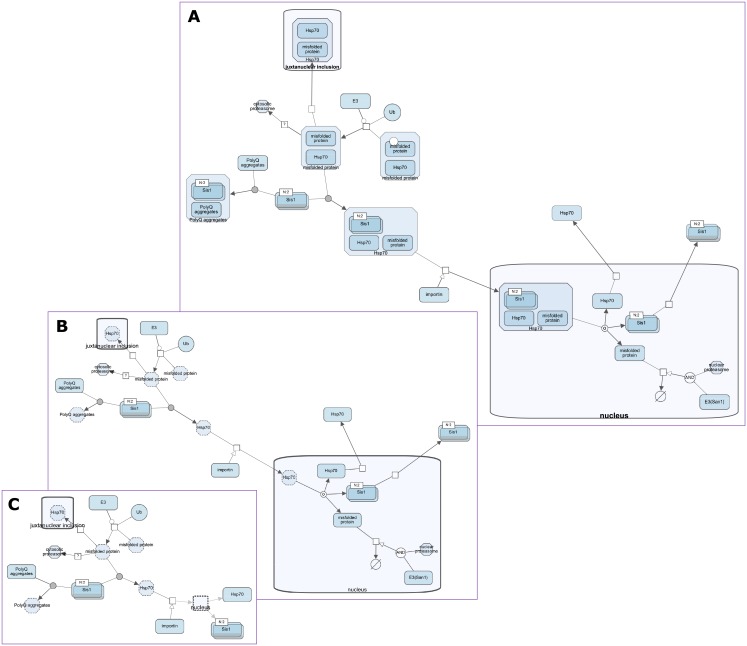
Expand-collapse example. Size of a biological map (A) is drastically reduced after all complexes (B) and then nucleus (C) are collapsed.

Expand-collapse related functionality is available in GitHub as a Cytoscape.js extension (https://github.com/iVis-at-Bilkent/cytoscape.js-expand-collapse) and on npm as a module (https://www.npmjs.com/package/cytoscape-expand-collapse). Fig 11 shows an example usage of this extension.

#### API for version 3.1.1

To initialize or subsequently set options of this extension defined and registered on a Cytoscape.js instance cy:

 cy.expandCollapse(options)

To get a handle to the extension instance after initialization:

 var api = cy.expandCollapse(‘get’)

Once initialized, following functions can be applied on an instance. These functions return a handle to options parameter to apply during a particular event unlike the function above:

- Collapse specified nodes, extending options with given parameters:
   api.collapse(nodes, options)
- Collapse specified nodes recursively, extending options with given parameters:
   api.collapseRecursively(nodes, options)
- Collapse all nodes in graph recursively, extending options with given parameters:
   api.collapseAll(options)
- Expand specified nodes, extending options with given parameters:
   api.expand(nodes, options)
- Expand specified nodes recursively, extending options with given parameters:
   api.expandRecursively(nodes, options)
- Expand all nodes in graph recursively, extending options with given parameters:
   api.expandAll(options)
- Return whether specified node is expandable (i.e., collapsed):
   api.isExpandable(node)
- Return whether specified node is collapsible (i.e., expanded):
   api.isCollapsible(node)
- Return expandable ones inside specified nodes (if nodes parameter is not specified consider all nodes):
   api.expandableNodes(nodes)
- Return collapsible ones inside specified nodes (if nodes parameter is not specified consider all nodes):
   api.collapsibleNodes(nodes)
- Reset the options to the given parameter:
   api.setOptions(options)
- Set value of the option given by the name to the specified value:
   api.setOption(name, value)
- Return children of the specified collapsed node, which are removed during collapse operation. Returned value includes both nodes and edges; use selector to get only nodes or only edges:
   api.getCollapsedChildren(node)
- Return collapsed children recursively, including nested ones. Returned value includes both nodes and edges; use selector to get only nodes or only edges:
   api.getCollapsedChildrenRecursively(node)
- Return collapsed children of all collapsed nodes recursively. Returned value includes both nodes and edges; use selector to get only nodes or only edges:
   api.getAllCollapsedChildrenRecursively(node)
- Force the visual cue to be cleared. It is to be called in exceptional cases:
   api.clearVisualCue()

#### Events

Following events are triggered at specified points during expand-collapse operations:

- Before a node is collapsed:
   cy.nodes().on(“expandcollapse.beforecollapse”,
    function(event) { var node = this; … })
- After a node is collapsed:
   cy.nodes().on(“expandcollapse.aftercollapse”,
    function(event) { var node = this; … })
- Before a node is expanded:
   cy.nodes().on(“expandcollapse.beforeexpand”,
    function(event) { var node = this; … })
- After a node is expanded:
   cy.nodes().on(“expandcollapse.afterexpand”,
    function(event) { var node = this; … })

#### Customization

The expand-collapse library is highly customizable. The customizable options should be provided as a JSON object during initialization of the library. If no options are provided, then default options are used. The list of all customizable options, their explanations and default values are given below:

- layoutBy: this option is used to specify whether or not nodes are rearranged (apply layout) after expand/collapse operations. The default value is null (i.e., nodes are not rearranged after expand/collapse) We recommend the use of cose-bilkent layout with randomize: false to preserve their mental map upon expand/collapse.
- fisheye: whether to perform fisheye based translation of neighboring nodes after expand/collapse. Value can be either a boolean or a function, which returns a boolean value dynamically. The default value of this option is true.
- animate: whether to animate on drawing changes. Value can be either a boolean or a function, which returns a boolean value dynamically. The default value of this option is true.
- undoable: whether to make expand/collapse operations undoable or not. The default value of this option is true. *Note:* requires undo-redo extension.
- cueEnabled: whether cues are enabled or not. The default value of this option is true.
- expandCollapseCuePosition: specifies the position of cues. The default value of this option is top-left. A function can be specified to calculate the position of cue per node as well. This function needs to return a position object.
- expandCollapseCueSize: this option is used to specify the size of expand/collapse cues. The default value is 12.
- expandCollapseCueLineSize: default cue icon for expand (collapse) operation is a plus (minus) sign. This option is used to specify the width of lines which are used for drawing default icons. The default value is 8.
- expandCueImage: image of expand icon. The default value is undefined (in this case, a plus icon is drawn).
- collapseCueImage: image of collapse icon. The default value is undefined (in this case, a minus icon is drawn).
- expandCollapseCueSensitivity: sensitivity of expand-collapse cues for hit-testing. The default value is 1.

### Hide-show

**Fig 12:**
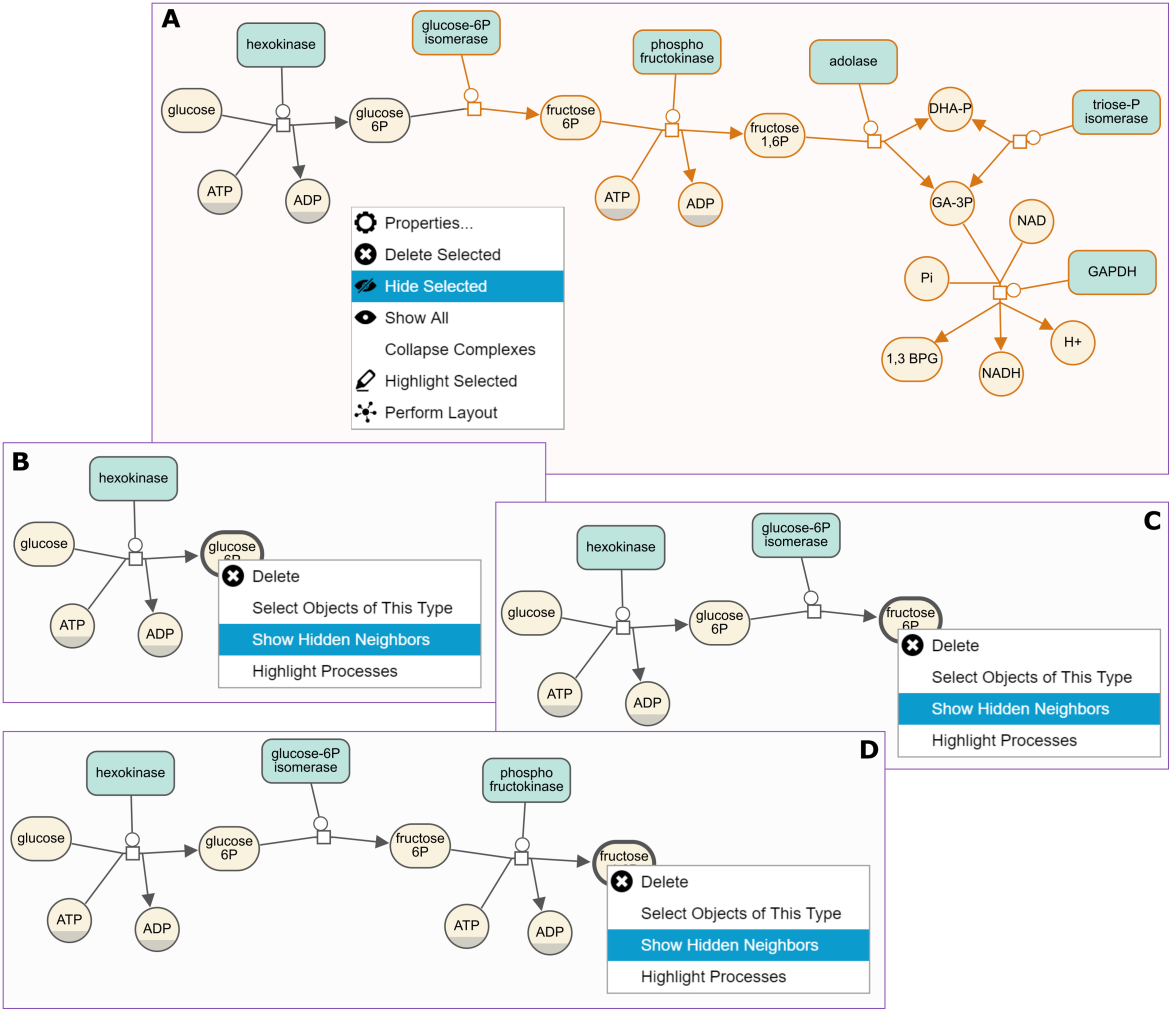
Hide-show example. First the user chooses to focus on a single process in an SBGN map (A,B) Then, the hidden details are gradually shown to assist in analysis (C,D).

Hide-show related functionality is available in GitHub as a Cytoscape.js extension (https://github.com/iVis-at-Bilkent/cytoscape.js-view-utilities) and on npm as a module (https://www.npmjs.com/package/cytoscape-view-utilities). Fig 12 shows an example usage of this extension.

#### API for version 2.1.0

To initialize the extension with options or override default options subsequently, use:

 var instance = cy.viewUtilities(options)

Once an instance is initialized, following functions can be invoked:

- Hide specified elements:
   instance.hide(eles)
- Unhide specified elements:
   instance.show(eles)
- Highlight specified elements and unhighlight others at first use:
   instance.highlight(eles)
- Unhighlight specified elements:
   instance.unhighlight(eles)
- Highlight specified elements’ neighborhood and unhighlights others at first use:
   instance.highlightNeighbors(eles) Alias:
   instance.highlightNeighbours(eles)
- Unhighlight specified elements and their neighbors:
   instance.unhighlightNeighbors(eles) Alias:
   instance.unhighlightNeighbours(eles)
- Remove highlights and unhighlights from specified elements. If the eles parameter is not specified, sets it to cy.elements():
   instance.removeHighlights(eles)

#### Customization

The hide-show library is highly customizable. The customizable options should be provided as a JSON object during initialization of the library. If no options are provided, then the default options are used. The list of all customizable options, their explanations and default values are given below:

- Style of highlighted/unhighlighted nodes and edges can be set as follows:
  node: {
   highlighted: {}, // style for when nodes are highlighted
   unhighlighted: { // style for when nodes are unhighlighted
    ‘opacity’: 0.3 // set opacity of unhighlighted nodes
   }
  },
  edge: {
   highlighted: {}, // style for when edges are highlighted
   unhighlighted: { // style for when edges are unhighlighted
    'opacity': 0.3 // set opacity of unhighlighted edges
   }
  }
  By default, the opacity is set to 0.3 when nodes/edges are unhighlighted.
- setVisibilityOnHide: whether to set visibility parameter of elements on hide/show. The default value is false.
- setDisplayOnHide: whether to set display parameter of elements on hide/show. The default value is true.
- neighbor: the vaule of this option should be a function, which returns neighbors of a given node. By default, no neigbours are returned.
- neighborSelectTime: users can select neighbors of a node interactively by presing Shift key and tapholding on that node. This option specifies the time required to taphold on a node to select desired neighbors. The default value is 500 *ms*.

## Conclusion

In this paper, we presented new specialized algorithms and readily customizable libraries for managing complexity in visual analysis of large networks. With these methods and libraries, we hope that tool developers will be more equipped in dealing with this challenge in network analysis software.

## References

[1] Khan N, Yaqoob I, Hashem IAT, Inayat Z, Ali M, Kamaleldin W, et al Big Data: Survey, Technologies, Opportunities, and Challenges. The Scientific World Journal. 2014;p. 712826 doi: 10.1155/2014/712826 25136682 10.1155/2014/712826 PMC4127205

[2] Dogrusoz U, Genc B. A multi-graph approach to complexity management in interactive graph visualization. Computers & Graphics. 2006;30(1):86–97. doi: 10.1016/j.cag.2005.10.015

[3] Sari M, Bahceci I, Dogrusoz U, Sumer SO, Aksoy BA, Babur O, et al SBGNViz: a tool for visualization and complexity management of SBGN process description maps. PLoS ONE. 2015;10(6):e0128985 doi: 10.1371/journal.pone.0128985 26030594 10.1371/journal.pone.0128985 PMC4451519

[4] Franz M, Lopes CT, Huck G, Dong Y, Sumer O, Bader GD. Cytoscape.js: a graph theory library for visualisation and analysis. Bioinformatics. 2016;32(2):309–311. doi: 10.1093/bioinformatics/btv557 26415722 10.1093/bioinformatics/btv557 PMC4708103

[5] Novère NL, Hucka M, Mi H, Moodie S, Schreiber F, Sorokin A, et al The systems biology graphical notation. Nature Biotechnology. 2009;27(8):735–741. doi: 10.1038/nbt.1558 19668183 10.1038/nbt.1558

[6] Battista GD, Eades P, Tamassia R, Tollis IG. Graph Drawing: Algorithms for the Visualization of Graphs. 1st ed Upper Saddle River, NJ, USA: Prentice Hall PTR; 1998.

[7] Fruchterman TMJ, Reingold EM. Graph Drawing by Force-directed Placement. Software Practice & Experience. 1991;21(11):1129–1164. doi: 10.1002/spe.4380211102

[8] Dogrusoz U, Giral E, Cetintas A, Civril A, Demir E. A compound graph layout algorithm for biological pathways In: Graph Drawing. Springer; 2005 p. 442–447.

[9] Herman I, Melançon G, Marshall MS. Graph Visualization and Navigation in Information Visualization: A Survey. IEEE Transactions on Visualization and Computer Graphics. 2000;6(1):24–43. doi: 10.1109/2945.841119

[10] Sarkar M, Brown MH. Graphical Fisheye Views. Communications of the ACM. 1994 12;37(12):73–83. doi: 10.1145/198366.198384

[11] Buchsbaum AL, Westbrook J. Maintaining hierarchical graph views. In: Proceedings of the Eleventh Annual ACM-SIAM Symposium on Discrete Algorithms, January 9-11, 2000, San Francisco, CA, USA.; 2000. p. 566–575.

[12] Misue K, Eades P, Lai W, Sugiyama K. Layout adjustment and the mental map. Journal of Visual Languages and Computing. 1995;6(2):183–210. doi: 10.1006/jvlc.1995.1010

[13] Storey MAD, Fracchia FD, Müller HA. Customizing a Fisheye View Algorithm to Preserve the Mental Map. Journal of Visual Languages and Computing. 1999;10:245–267. doi: 10.1006/jvlc.1999.0124

[14] Shannon P, Markiel A, Ozier O, Baliga N, Wang J, Ramage D, et al Cytoscape: a software environment for integrated models of biomolecular interaction networks. Genome Research. 2003;13(11):2498–2504. doi: 10.1101/gr.1239303 14597658 10.1101/gr.1239303 PMC403769

[15] Bastian M, Heymann S, Jacomy M. Gephi: An Open Source Software for Exploring and Manipulating Networks. In: International AAAI Conference on Weblogs and Social Media; 2009.

[16] Dogrusoz U, Erson EZ, Giral E, Demir E, Babur O, Cetintas A, et al PATIKAweb: a Web interface for analyzing biological pathways through advanced querying and visualization. Bioinformatics. 2006;22(3):374–375. doi: 10.1093/bioinformatics/bti776 16287939 10.1093/bioinformatics/bti776

[17] Tom Sawyer Perspectives;. Accessed: 2018-04-04. http://www.tomsawyer.com.

[18] Bostock M, Ogievetsky V, Heer J. D3 Data-Driven Documents. IEEE Transactions on Visualization and Computer Graphics. 2011;17(12):2301–2309. doi: 10.1109/TVCG.2011.185 22034350 10.1109/TVCG.2011.185

